# Identification of a Two-Gene (*PML-EPB41*) Signature With Independent Prognostic Value in Osteosarcoma

**DOI:** 10.3389/fonc.2019.01578

**Published:** 2020-01-24

**Authors:** Shengye Liu, Jiamei Liu, Xuechen Yu, Tao Shen, Qin Fu

**Affiliations:** ^1^Department of Spine and Joint Surgery, Shengjing Hospital of China Medical University, Shenyang, China; ^2^Department of Pathology, Shengjing Hospital of China Medical University, Shenyang, China; ^3^Hammer Health Sciences Center, Columbia University Medical Center, New York, NY, United States

**Keywords:** osteosarcoma, protein-protein interaction network, gene signature, prognostic prediction, survival analysis

## Abstract

**Background:** Osteosarcoma (OSA) is the most prevalent form of malignant bone cancer and it occurs predominantly in children and adolescents. OSA is associated with a poor prognosis and highest cause of cancer-related death. However, there are a few biomarkers that can serve as reasonable assessments of prognosis.

**Methods:** Gene expression profiling data were downloaded from dataset GSE39058 and GSE21257 from the Gene Expression Omnibus database as well as TARGET database. Bioinformatic analysis with data integration was conducted to discover the significant biomarkers for predicting prognosis. Verification was conducted by qPCR and western blot to measure the expression of genes.

**Results:** 733 seed genes were selected by combining the results of the expression profiling data with hub nodes in a human protein-protein interaction network with their gene functional enrichment categories identified. Following by Cox proportional risk regression modeling, a 2-gene (*PML-EPB41*) signature was developed for prognostic prediction of patients with OSA. Patients in the high-risk group had significantly poorer survival outcomes than in the low-risk group. Finally, the signature was validated and analyzed by the external dataset along with Kaplan–Meier survival analysis as well as biological experiment. A molecular gene model was built to serve as an innovative predictor of prognosis for patients with OSA.

**Conclusion:** Our findings define novel biomarkers for OSA prognosis, which will possibly aid in the discovery of novel therapeutic targets with clinical applications.

## Introduction

Osteosarcoma (OSA) is an aggressive malignant neoplasm that occurs around the metaphysis of tubular long bones. It exhibits osteoblastic differentiation and produces malignant immature osteoid (1, 2), which causes a painful health burden and potentially fatal complications. OSA occurs primarily in children and adolescents: more than 26,400 new cases are diagnosed each year and the disease is frequently associated with poor clinical outcomes (3). Chemotherapeutic and radiation treatments cannot efficiently treat OSA and their varied efficacies may be due to individual differences and patient-specific factors. Moreover, the toxicity of some chemotherapeutic drugs cannot be avoided (4, 5), which adds to the pain and burden of disease associated with OSA. Therefore, it is imperative that clinicians and patients understand the best options for the treatment of OSA and prevention of undesirable complications. Over the past two decades, the survival rate of patients with OSA has not substantially improved. Many studies of the molecular mechanism of OSA have identified several oncogenes and tumor suppressors that play significant roles in the tumorigenesis of OSA (6, 7). However, no generally recognized biomarkers have been established to facilitate the comprehensive management and the prognostic prediction of patients with OSA, which might help overcome the poor survival rate of OSA. With the rapid development of high-throughput sequencing and improvements in data analysis techniques, the discovery of cancer-related genes and the identification of clustering models can rely on bioinformatics and data integration. Recently, many biomarkers, including MYC and SP1, have been verified to be correlated with the progression and clinical diagnosis of OSA (8, 9). Additionally, the identification of tumor biomarkers and the discovery of gene functions are the focuses of many studies (10, 11). However, few research has focused on establishing specific gene signatures that can accurately guide patient therapy when predicting the outcomes of OSA. Thus, identifying tumor markers or constructing feature gene models are still the focus of much research and study.

The purpose of this study was to explore the biological significance of certain gene candidates for predicting prognosis in OSA. We focused on analyzing gene expression profiles in a Gene Expression Omnibus (GEO) dataset combined with a human protein-protein interaction (PPI) network to identify the seed genes that affect the prognosis of patients with OSA. Using functional enrichment, Cox proportional hazards regression modeling, and Kaplan–Meier survival analysis, we established a 2-gene prognostic signature that can serve as a model for predicting prognosis in OSA. Finally, the consistency and availability of our findings were confirmed by an external GEO dataset and TARGET database. Based on the conclusion above, experimental validation was conducted on human normal osteoblast and OSA cell line. The 2-gene signature can provide new insights for monitoring the prognostic status of patients with OSA.

## Materials and Methods

### Data Source

Gene expression profiling data encompassing OSA were downloaded from GEO (12) in the National Center for Biotechnology Information database. We used the mRNA of internal testing dataset GSE39058, including 2 subsets: GSE39055 and GSE39057 (13) (platform: GPL14951 Illumina HumanHT-12 WG-DASL V4.0 R2 expression beadchip). Expression profiling contained 29,377 gene probes and 47 samples, including 37 independent OSA biopsy samples and 5 paired samples (5 OSA biopsy samples and 5 surgical resection specimens); patient clinical information and survival time data were also included. GSE21257 (14) was used as an external dataset of verification (platform: GPL10295 Illumina human-6 v2.0 expression beadchip). This dataset included 53 samples of OSA and follow-up information. TARGET dataset including 84 samples was also applied for further validation.

### Identification of Seed Genes

The degree of variation of each sample gene was measured by the CV, which was calculated according to the following formula: $CV\;=\;\frac{SD}{MN}\times 100\%$, where SD refers to the standard deviation, and MN is the mean. The genes for which the CV was larger than 20% were selected for further analysis as the seed genes of OSA.

### Unsupervised Hierarchical Clustering

Based on the seed genes obtained in the previous step, 47 samples underwent unsupervised hierarchical clustering using the hclust function in R and the Euclidean distance measurement. The heatmap was completed with the pheatmap package, which revealed the gene expression levels and clinical information of the samples.

### PPI Network Construction

Five PPI network databases, including the Human Protein Reference Database (15), the Database of Interacting Proteins (16), the Biomolecular Interaction Network Database (17), IntAc (18) and the Molecular INTeraction Database (19), were integrated to develop a PPI network. Seed genes were entered into the network and became the key nodes. Each node and its closest neighbor genes were then extracted to construct a sub-network of candidate genes.

### Functional Enrichment Analysis

A web server for the functional interpretation of Gene lists, g: Profiler (20) (http://biit.cs.ut.ee/gprofiler/), was used to carry out the enrichment analysis of Gene ontologies, Kyoto Encyclopedia of Genes and Genomes pathway analysis, and human protein profiling for these candidate genes. EnrichmentMap (21), a Cytoscape plugin, allowed for visualization of the gene enrichment analysis results, which contributed to a better understanding of the gene functional enrichment categories.

### Construction of the 2-Gene Signature

First, univariate Cox regression analysis for each candidate gene was used to identify genes that significantly (*p* < 0.01) influenced the prognosis of OSA. Then, we enrolled the genes into the multivariate Cox regression analysis to determine the independent prognostic factors that are not affected by covariates such as age, sex, and recurrence. The Cox proportional risk regression model was constructed by the coxph function in survival R package (Tables 1, 2). Next, the regression coefficients and expression values of the genes that significantly influenced the prognosis of OSA were used to establish the PI, which was calculated according to the following formula: *PI* = ∑ *coef* (*gene*_*i*_) × exp (*gene*_*i*_). Patients were divided into high-risk and low-risk groups on the basis of median PI value. The significance of the difference between the 2 groups was determined by the log-rank test, and Kaplan–Meier survival curves were performed to determine the survival state of OSA patients. The ROC curves of the PI signature and the genes in the signature were compared by using timeROC function in R. We conducted single-sample GSEA analysis by GSVA function. The ssGSEA score corresponding to each function was obtained according to the expression matrix. The correlation between these functions and RiskScores was further calculated to realize potential regulatory pathways of PI.

**Table 1:** Univariate and multivariate cox survival analysis in the training GSE39058 and two external datasets (TARGET database and GSE21257).

**Variables**	**Univariate analysis**			**Multivariable analysis**		
	**HR**	**95% CI of HR**	***P*-value**	**HR**	**95% CI of HR**	***P*-value**
**GSE39058**						
2-gene risk score						
Risk score (high/low)	6.631	1.43–30.75	1.56E-02	8.991	1.74–46.21	8.50E-03
Age	0.997	0.957–1.039	0.922	0.981	0.942–1.022	0.371
Gender (male/female)	1.045	0.329–3.308	0.941	0.576	0.171–1.947	0.375
**TARGET datasets**						
2-gene risk score						
Risk score (high/low)	2.944	1.33–6.51	7.00E-03	3.118	1.393–6.977	5.65E-03
Age	0.999	0.999–1.001	0.81	1	0.999–1.000	0.809
Gender (male/female)	0.868	0.624–1.208	0.402	0.977	0.646–1.479	0.915
Metastatic vs. non-metastatic	4.74	2.27–9.89	3.41E-05	5.09	2.416–10.73	1.88E-05
**GSE21257**						
7-gene risk score						
Risk score (high/low)	2.7	1.17–6.19	0.019	2.85	1.215–6.681	0.016
Age	0.999	0.996–1.004	0.957	1.002	0.998–1.005	0.319
Gender (male/female)	1.402	0.58–3.34	0.44	1.407	0.584–3.387	0.446

**Table 2:** Detailed sample information of internal and external datasets.

**Characteristics**		**GSE39058 (*n* = 42)**	**TARGET all datasets (*n* = 84)**	**GSE21257 (*n* = 53)**
Age (years)	<=18	21	66	35
	>18	21	18	18
Survival status	Living	30	55	30
	Dead	12	29	23
Gender	Female	20	37	19
	Male	22	47	34
Metastatic	Metastatic	20	21	34
	Non-metastatic	22	53	19

### Identification of Two Genes by Experimental Test

Human osteoblast cell line hFOB1.19 was maintained in DMEM/F12 medium at 34°C with 5% CO_2_ in a humidified atmosphere. Human osteosarcoma cell line MG-63 and SAOS-2 cells were cultured in MEM and Macoy'5A medium, respectively. Medium were all supplemented with a 10% FBS, 100 μgml streptomycin and 100 U/ml penicillin at 37°C with 5% CO_2_ in a humidified atmosphere. Quantification of gene and protein expression measurement is carried out by quantitative polymerase chain reaction and western blot as previously cited. The primer sequences were as follows: forward, 5′-ACCAACAACATCTTCTGCTCCAACC-3′ and reverse, 5′-CCGAGGCGTAGCACTTCATCC-3′ for PML; The primer sequences were as follows: forward, 5′-ACAGGTCCATGACTCCAGCTCAG-3′ and reverse, 5′-ACCAGAAGGCCACTAGAGCAGAC-3′ for EPB41; and forward, 5′-GTGAAGCAGGCATCTGAGGG-3′ and reverse, 5′-GCCGTATTCATTGTCATACCAGG-3′ for GAPDH. The EPB41 antibody (Cat. no. 13014-1-AP), PML antibody (Cat. no. 21041-1-AP) and GAPDH antibody (Cat. no. 60004-1-Ig) were obtained from Proteintech. All the presented data and results were processed using GraphPad Prism 7.02 software and expressed as mean ± standard deviation of at least three independent experiments. *T*-test was used to determine statistical significance. *P* < 0.05 or *P* < 0.01 were considered to indicate statistically significant differences.

## Results

### Identification of Seed Genes Based on the Coefficient of Variation in OSA

**Figure 1:**
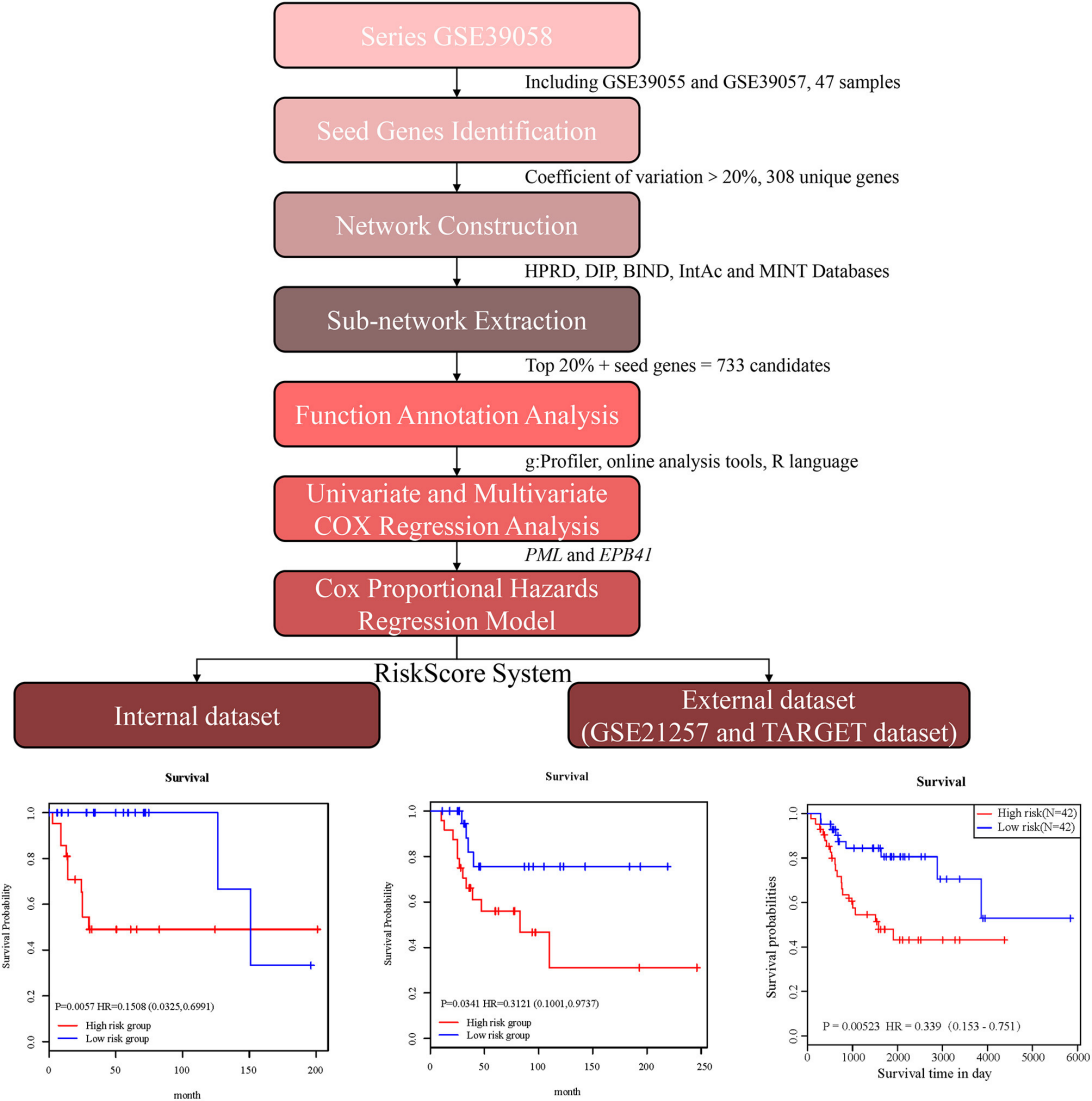
Schematic diagram for a multi-step strategy to identify 2-gene signature for the prognostic prediction of osteosarcoma.

**Figure 2:**
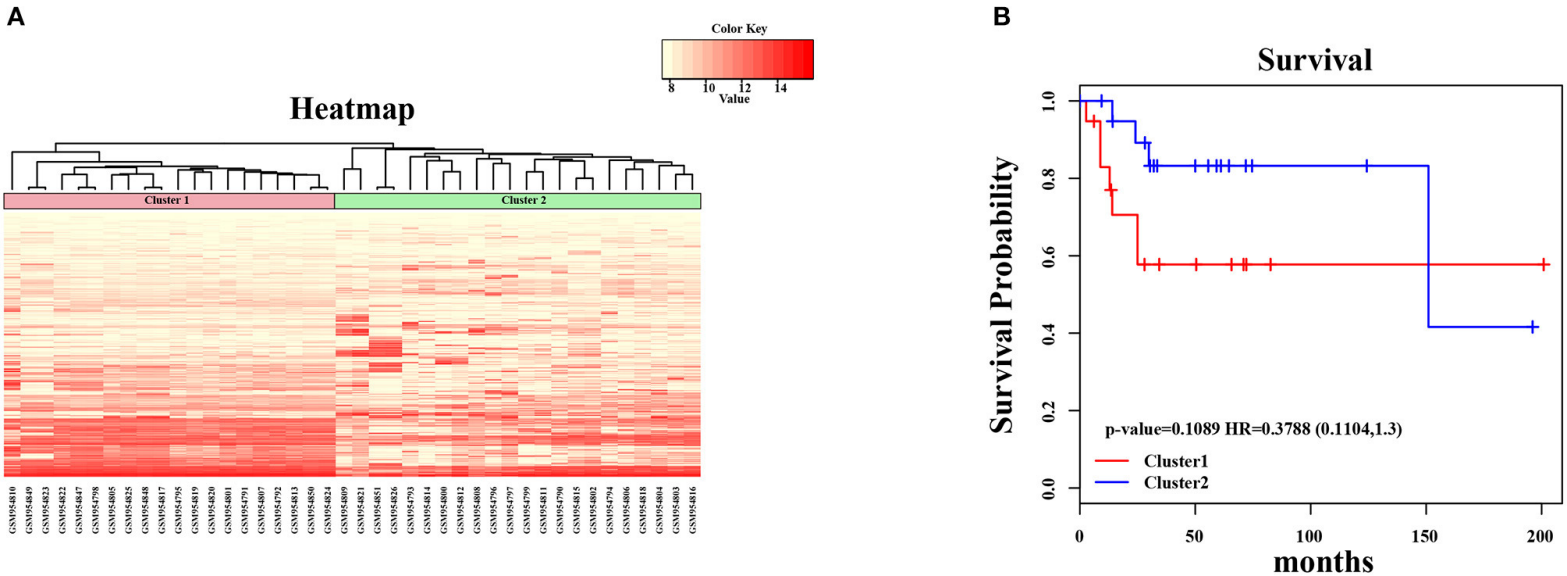
Unsupervised hierarchical clustering analysis for 2-genes. **(A)** The expression heatmap of seed genes in all OSA tumor samples. The horizontal axis above represents the samples, using Euclidean distance; the samples were grouped into two clusters (cluster 1 and cluster 2). **(B)** The Kaplan–Meier survival curves of two different clusters. There was no significant difference between two clusters (log-rank test *p* > 0.05).

In the present study, we established a 2-gene signature for the prognostic prediction of OSA (Figure 1). First, we used the GSE39058 dataset, which included 47 samples, as a training set. In this dataset, each patient sample included detailed clinicopathologic information and survival status. The coefficient of variation (CV) of each probe was calculated for all samples, and the probes with a CV >20% were considered to have the largest degree of variation among all OSA samples and were selected as the seed probes. Then, 309 probes were obtained and mapped to 308 unique genes. Next, we completed an unsupervised clustering analysis of the 42 samples by using expression profiling of the 309 probes obtained in the previous step. As shown in Figure 2A, the OSA samples were divided into 2 groups, and there were significant differences in gene expression levels between the groups. Survival analysis was then used to compare the outcomes of the groups: no significant difference was observed between them (log-rank test *p* ≥ 0.05) (Figure 2B).

### Network Construction and Sub-network Extraction Based on PPI Databases

**Figure 3:**
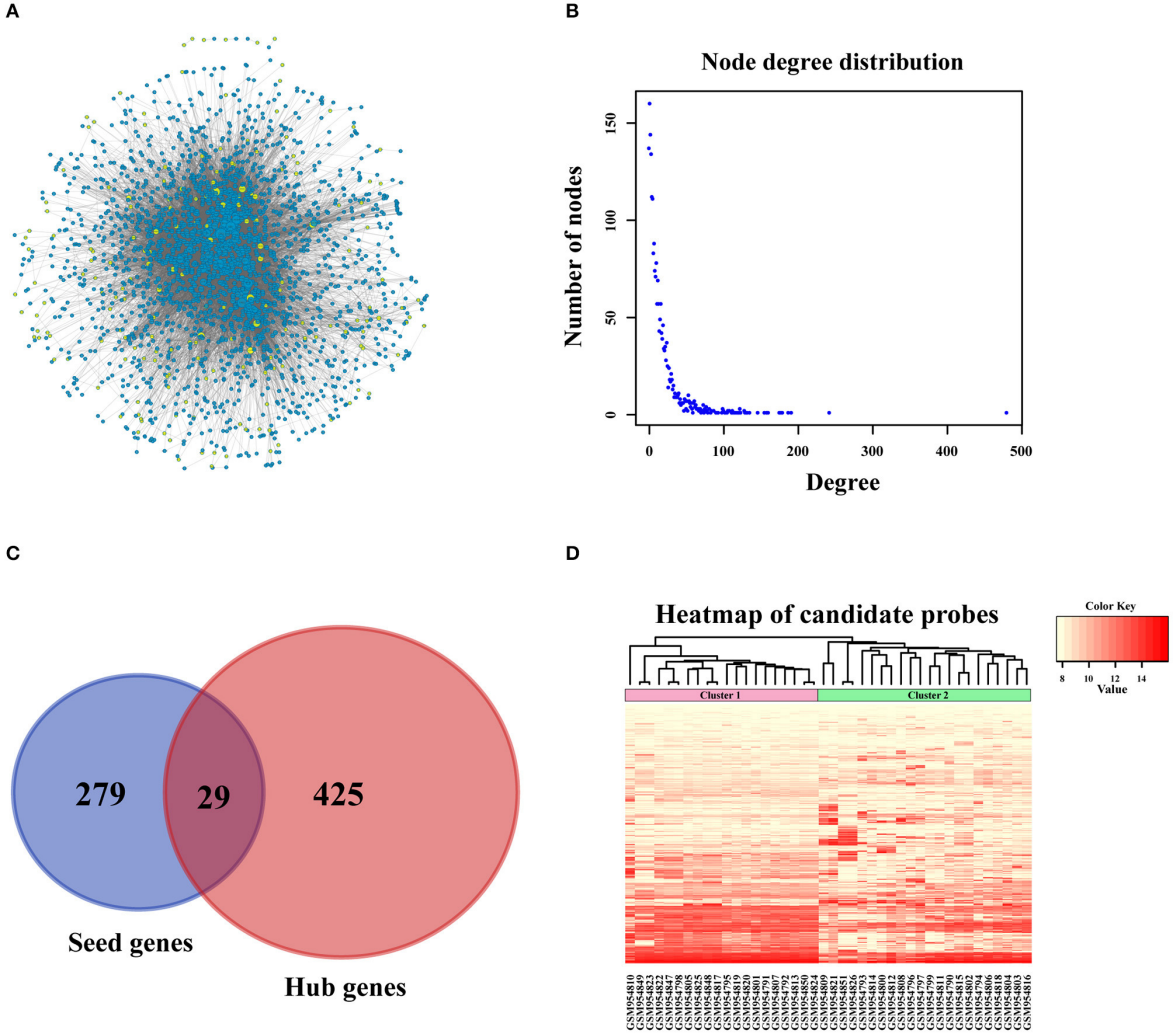
PPI network construction. **(A)** Sub-network of candidate genes. Three hundred and eight seed genes were subjected into PPI network and 192 nodes were obtained. The above nodes and its closest neighbor genes were extracted to construct sub-network containing 2,270 nodes. **(B)** The distribution of interacted nodes. **(C)** The venny gram of seed genes and hub genes. **(D)** The expression heatmap of all candidate genes.

In order to amplify potential candidate genes for further analysis, we integrated 5 human PPI databases, as mentioned in the Materials and Methods section. First, we constructed a background network that included 13,368 genes with 80,977 interaction pairs (Table S1). We then entered 308 seed genes into the network and identified 192 nodes. Each identified node and its closest neighbor genes were extracted to construct a sub-network containing 2,270 nodes (Figure 3A). As shown in Figure 3B, the distribution of interacting nodes was consistent with power-law distribution, which suggests the accuracy of the sub-network extraction.

Hub nodes, the larger-degree nodes in this network, may play significant roles in molecular progress. In this network, the largest node was UBC (degree = 481), which has been reported to be a target gene for the diagnosis and treatment of OSA (22). Moreover, many studies have proved that a mutation in the second largest node, TP53 (degree = 243), is closely linked with the development (23), prognosis (24), and tumor susceptibility (25) of OSA. The third largest gene, YWHAZ (degree = 192), has been shown to be a potential biomarker for the occurrence and treatment of OSA tumors and affects patient prognosis (26). Interestingly, none of these top 3 genes were screened for OSA in the previous step, which proved that PPI network construction for OSA-related genes was necessary. Second, we selected hub nodes from the network that ranked in the top 20%, which amounted to 454 genes (Table S2). Together with the above-mentioned 308 identified seed genes, we obtained a final total of 733 genes (Figure 3C) as the candidate genes for further analysis. These genes, which corresponded to 1,174 gene probes, were also used to divide the OSA samples into 2 clusters (Figure 3D), which is the same as the above results in Figure 2A.

### Functional Annotations of Candidate Genes Based on Bioinformatics Analysis

**Figure 4:**
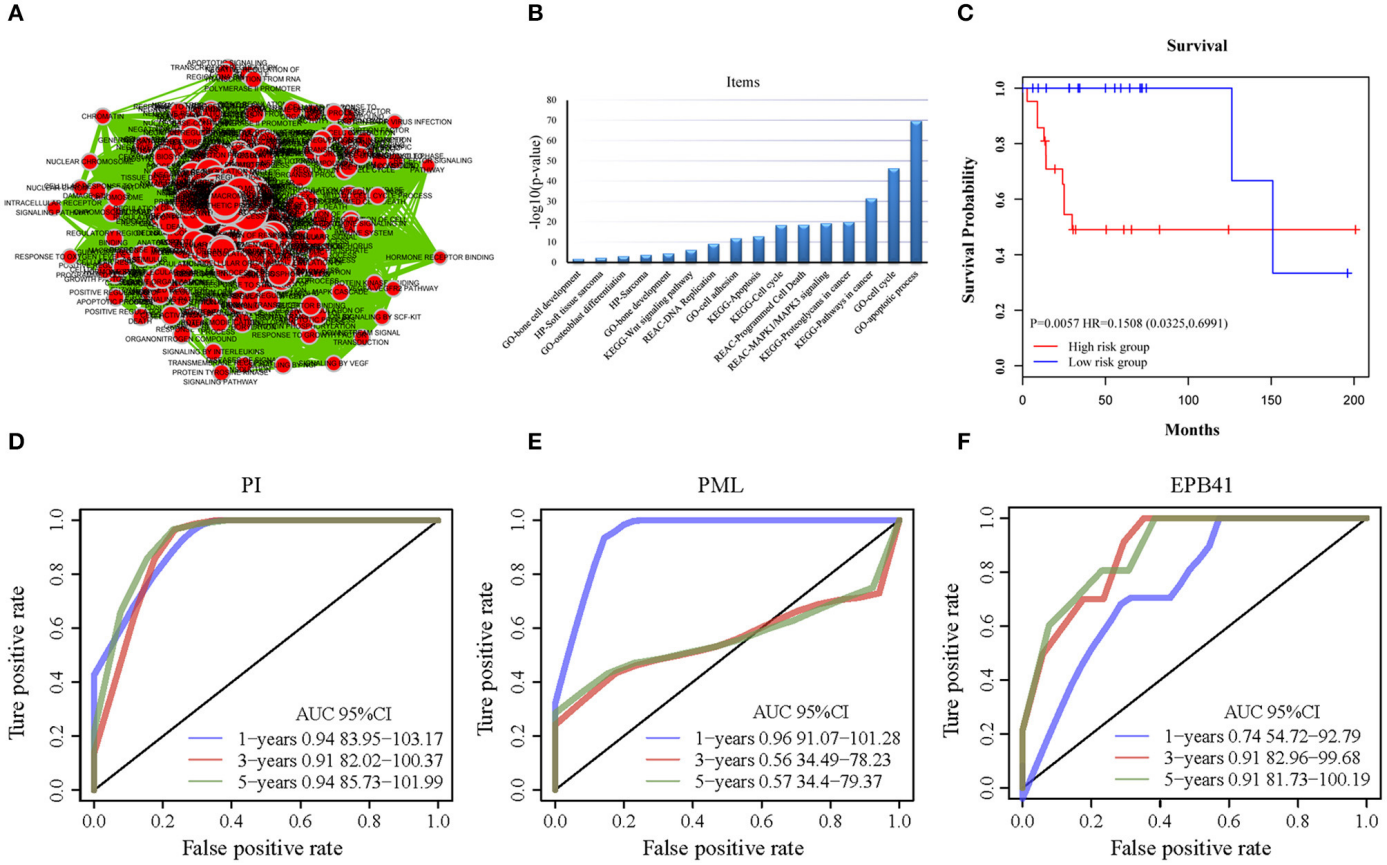
The functional analysis of candidate genes. **(A)** Visualization for functional enrichment results of 733 candidate genes by Cytoscape software. Each node represents an annotation term. **(B)** The results of g: profiler functional enrichment with prognostic candidate genes. Horizontal axis above represents annotation terms. Vertical axis represents the value of –log_10_ (*p*-value). **(C)** The Kaplan–Meier curves for overall survival of high- and low-risk groups of OSA in the internal training dataset and external validating dataset. The prognostic differences between the two groups in internal training dataset was significant (log-rank test *p* = 0.0057). **(D)** ROC of PML-EPB41 signature based on PI in training dataset samples. **(E)** ROC of PML signature in training dataset samples. **(F)** ROC of EPB41 signature in training dataset samples.

All of the 733 candidate genes may participate in the genesis and progression of OSA. Therefore, we used a comprehensive bioinformatics analysis to perform a gene functional analysis. Using an online tool (g: Profiler), we completed functional annotation for the 733 candidates; the EnrichmentMap tool of Cytoscape software was used to visualize the results (Figure 4A). The findings indicated that the genes were associated with many biological and pathological processes (Table S3), such as the development and differentiation of bone tissue, bone development, bone cell development, osteoclast differentiation, and the acute bone marrow leukemia pathway, as well as bone marrow and hematopoietic cells involved in human protein mapping. Representative results are shown in Figure 4B and indicate that the abnormality of these genes may lead to lesions of the bone tissue (Table S4). There were many familiar pathways that were closely connected with bone derived sarcoma formation, such as soft tissue sarcoma, osteoclast differentiation, apoptosis process, cell cycle, cell adhesion, etc. Wnt signaling pathway and MAPK1/MAPK3 signaling pathway have been widely reported in previous studies regarding OSA. Due to the diversity of proteoglycans in cancer, its influence on OSA deserves further study in the future.

### Establishment of a 2-Gene Signature of OSA

We completed expression profiling of the corresponding probes for the 733 genes, and we obtained expression values for a total of 1,174 probes. Patient clinical information was collected for 42 biopsy samples, including age, sex, disease recurrence, survival time, and survival status. Cox regression analysis was then applied on the basis of the probes.

First, the univariate Cox regression analysis was performed for each probe, and 9 probes were identified (Table 3) that significantly influenced survival (*p* < 0.01). The 9 probes were then introduced into the multivariate Cox regression analysis. Age, sex, and recurrence were used as covariates. We found 2 independent prognostic candidate genes of patient survival that were not affected by these covariate factors: ILMN_1811588 and ILMN_1663786, which corresponded to genes *PML* and *EPB41*, respectively (Table 4).

**Table 3:** Result of univariate Cox regression analysis.

**Probe**	**Gene**	***P*-value**	****β****
ILMN_2253720	*DHX30*	0.0020	3.3967
ILMN_1663786	*EPB41*	0.0030	0.9986
ILMN_1760542	*PSMA1*	0.0033	−8.5203
ILMN_1811588	*PML*	0.0036	2.7934
ILMN_1747146	*TSG101*	0.0048	−2.5886
ILMN_2241679	*UBE2D3*	0.0052	−12.0814
ILMN_3250923	*CREB1*	0.0061	1.6714
ILMN_1704557	*RPS6KB1*	0.0066	1.1951
ILMN_1765189	*PTK2B*	0.0074	−8.2439

**Table 4:** Result of multivariate Cox regression analysis.

**Probe**	**Gene**	**Covariate**	***P*-value**	**β**
ILMN_1811588	*PML*	ILMN_1811588	0.0066	3.8581
		Age	0.2100	−0.0515
		Gender	0.9500	−0.0458
		Recurrence	1.0000	21.9600
ILMN_1663786	*EPB41*	ILMN_1663786	0.0082	0.9718
		Age	0.3700	0.0389
		Gender	0.6000	−0.4248
		Recurrence	1.0000	22.8354

With the expression values of these 2 genes and multivariate Cox regression coefficients, we constructed the prognostic index (PI) as follows: *PI*(3.8581) × *PML expression value*+(0.9718) × *EPB*41 *expression value*. Patients were divided into 2 groups according to the median PI value. The survival analysis and the log-rank test revealed a significant difference in survival outcomes between the groups [log-rank test *p* = 0.0057, hazard ratio = 0.1508, 95% confidence interval = (0.0325, 0.6991)] (Figure 4C). We suggest that these 2 genes could be used as prognostic risk markers for OSA. Furthermore, we conducted ROC analysis on prognosis classification of PML, EPB41 and PML-EPB41 signature through timeROC function in the training dataset, and the prognostic AUC of 1, 3, and 5 years were analyzed, respectively. It was found in Figures 4D–F that the AUC of 1, 3, and 5 years of PI was above 0.91. Although the 1-year AUC of PML gene was above 0.96, its 3- and 5-year AUC were not ideal. The overall AUC value of EPB41 was no better than that of the PML-EPB41 signature. Therefore, it indicated that the PML-EPB41 signature constructed by us is more effective than the two genes signature alone.

**Figure 5:**
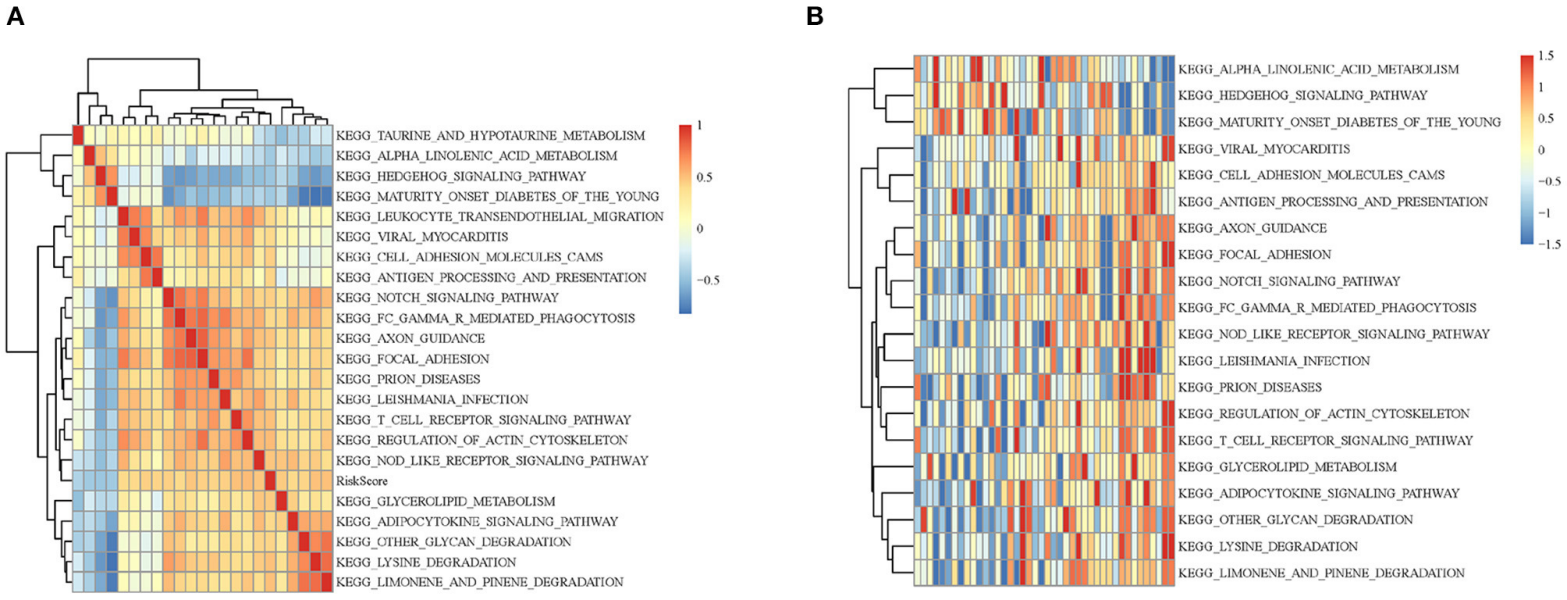
**(A)** Clustering analysis between KEGG pathway whose RiskScore correlation >0.4 and RiskScore correlation coefficients. **(B)** The relationship between the change of ssGSEA score and KEGG pathway RiskScore in each sample. The horizontal axis represents each sample, with risk scores rising from left to right.

In order to observe the relationship between risk scores of different samples and biological functions, we selected the corresponding gene expression profiles of these samples and used GSVA function in R to conduct single-sample GSEA analysis. By calculating the scores of different functions in each sample, the ssGSEA score of each sample was obtained. The correlation among these functions and the risk score was further computed, and the function whose correlation was >0.4 were selected. It can be seen that a few of them were negatively correlated with the sample RiskScore (Figure 5A), while most of them were positively correlated with the RiskScore. 20 KEGG pathways that were most correlated with more than 0.4 were selected for cluster analysis based on their enrichment scores. As Figure 5B illustrated, among the 20 pathways, metabolism-related pathways, decreased with the RiskScore increasing, such as HEDGEHOG SIGNALING PATHWAY and ALPHA LINOLENIC ACID METABOLISM. Immune-related pathways, such as NOTCH SIGNALING PATHWAY and T CELL RECEPTOR SIGNALING PATHWAY, increased as RiskScore rose, which also suggested that the dysregulation of these pathways was closely related to the progression of tumor.

### External Dataset Validation of the 2-Gene Signature in OSA

**Figure 6:**
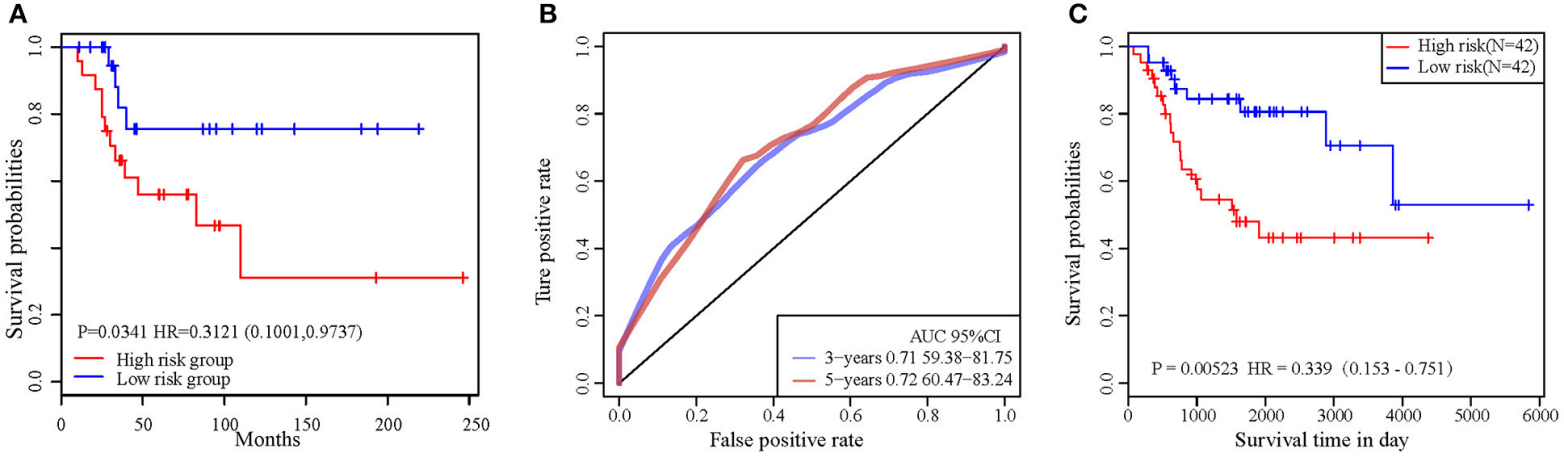
**(A)** The prognostic differences between the two groups in external validating dataset was significant (log-rank test *p* = 0.0341). **(B)** ROC curve in external validation TARGET dataset. **(C)** 2-gene signature KM curve in external validation TARGET dataset.

In order to validate the accuracy and prognostic value of the signature, an external validating dataset, GSE21257, was applied. This dataset included 53 samples of OSA, as well as follow-up information. Based on the expression levels of *PML* and *EPB41*, the PI was constructed as described above. The patients were also divided into high-risk and low-risk groups using the median PI value. From the results of the Kaplan–Meier survival curves, we found that there were significant prognostic differences between the 2 groups (log-rank test *p* = 0.0341) (Figure 6A), indicating that this 2-gene signature was indeed a key prognostic predictor for patients with OSA.

In addition, we downloaded the 84 OSA related expression profile data with clinical information from the TARGET database. The same method above was used to verify the results in the TARGET dataset, indicating a significant difference in prognosis between the two groups (*p* = 0.0052). TimeROC function was used to conduct ROC analysis on the prognosis classification of PI. The 3- and 5-year prognostic classification efficiency was obtained. As shown in Figure 6B, it could be concluded that the signature had a large AUC, with 0.71 in 3-year and 0.72 in 5-year, respectively. From the results of the Kaplan–Meier survival curves in Figure 6C, it was found that there were significant prognostic differences between the 2 groups in TARGET dataset (log-rank test *p* = 0.00523). The result above indicated two-gene signature were capable of influence OSA prognosis.

### Biological Experimental Validation of PML-EPB41 Expression in Cell Lines

**Figure 7:**
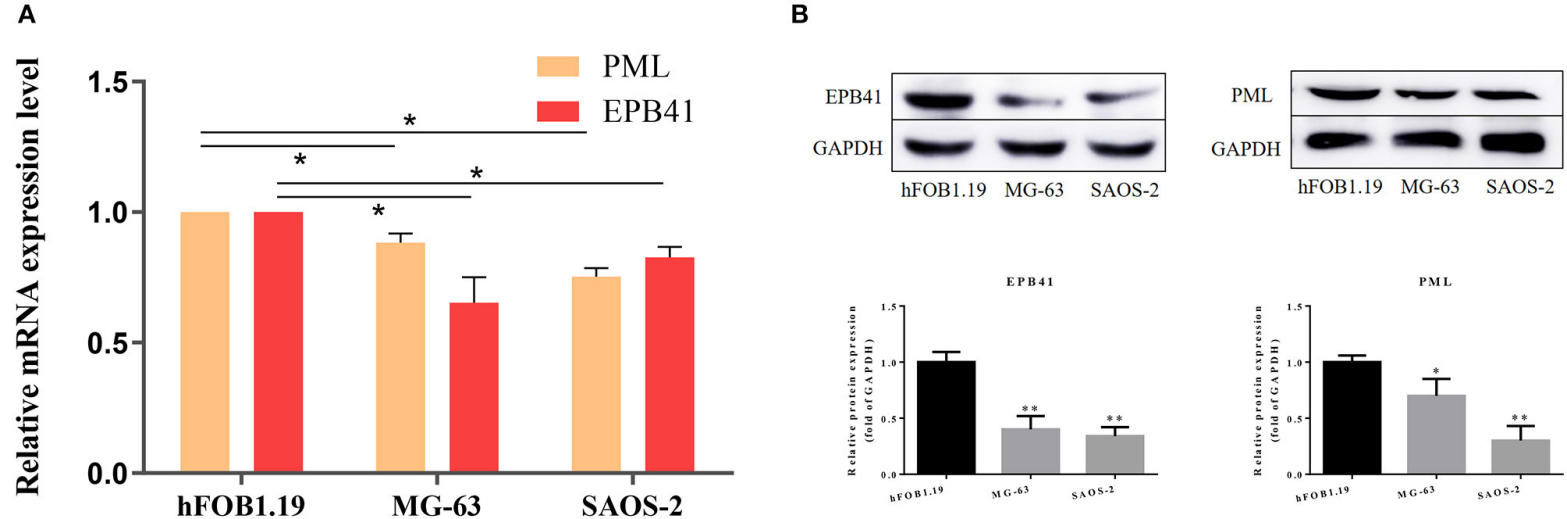
**(A)** Relative mRNA expression level of PML and EPB41 in three cell lines are conducted by quantitative polymerase chain reaction. Data are presented as the mean ± SD. ^*^P < 0.05 vs. hFOB1.19. **(B)** Expression of EPB41 and PML by western blot. Protein levels were statistically evaluated in columns. Measurements were in triplicate and data were presented as the mean ± SD. ^**^P < 0.01 vs. hFOB1.19.

By quantitative polymerase chain reaction, relative mRNA expression level of PML and EPB41 were measured among hFOB1.19, MG-63 and SAOS-2. As Figure 7 illustrated, the mRNA expression level of PML and EPB41 decreased significantly in MG-63 and SAOS-2 cells compared with hFOB1.19 cells (^*^P < 0.05). Furthermore, protein expression level of PML and EPB41 were also down-regulated by western blot in OSA cell lines (^**^P < 0.01).

## Discussion

In the present study, we first obtained 308 candidate seed genes by screening the gene expression profiling of OSA in a testing dataset. These genes were closely associated with the occurrence, development, disease subtype, prognosis, and differential response to therapeutic treatment of OSA. In order to amplify the seed genes for a more comprehensive analysis, 454 genes were further screened through a human PPI network as the hub nodes that were thought to be potential OSA-related genes. These genes were selected as candidate genes along with the 308 differential genes. The candidate genes were all associated with biological processes such as bone cell development and cancer pathways. Based on the construction of a univariate and multivariate Cox proportional risk regression model, we successfully developed a 2-gene (*PML-EPB41*) signature for predicting the prognosis of patients with OSA. An external data set was applied to verify the feasibility and reliability of the *PML-EPB41* signature. It was verified that the 2-gene clustering model effectively classified patients from the selected dataset into high-risk and low-risk groups with significant differences observed in survival time and reoccurrence risk according to Kaplan–Meier survival analysis in both testing and external datasets.

In previous studies, bioinformatics analysis was used to identify potential target genes, transcription factors, and gene functions in OSA. Additional analysis was conducted to identify the differential expressed genes (DEGs) and related potential biological process of OSA progress, in order to provide better guidance and clinical treatment options for patients (8–10, 27, 28). DEGs and their biological functions were identified by the integration of several GEO data sets (29). He et al. used an additional independent microarray dataset to verify the OSA-related pathway enrichment modules (30). Moreover, integrated whole-genome analysis was completed by identifying gene expressions and genomic aberrations in OSA, including single-nucleotide polymorphisms and copy number variants (11). However, all of these studies remain in the primary stage of differential gene screening and biological process enrichment. A specific and efficient way to predict the prognosis of OSA has not been confirmed. This study presents, for the first time, the 2-gene (*PML-EPB41*) signature that was built on the basis of the patient outcomes, which reflects overall patient prognosis. The 2-gene signature can be used to predict risks and prognosis of OSA and is not impacted by patient age, sex, or relapse.

The verification by independent dataset proved that *PML* and *EPB41* are indeed the key genes that affect the prognosis of OSA. The nuclear scaffold protein promyelocytic leukemia gene (*PML*) has a dual role in cancer: it can act as the downstream target of oncogenic RAS and it can promote tumorigenesis (31). *PML* is a pro-apoptosis gene that can be indirectly suppressed by cisplatin-based systemic chemotherapy in non-small cell lung carcinoma (32). Additionally, it is suggested that it has a potential role in immune-modulatory approaches for treating lung cancer with aberrant *PML* degradation (33). Silencing of *PML* also inhibits cell proliferation and induces DNA damage in cultured ovarian cancer cells (34). In OSA, *PML* has significantly different expression levels among OSA cell lines (35), and, as a suppressor gene in an OSA cell line, *PML* has been demonstrated to physically and functionally interact with oncogene MDM2 to regulate the biological behavior of tumor cells (36). The *EPB41* gene encodes Erythrocyte Membrane Protein Band 4.1. *EPB41* has been identified to function as a tumor suppressor in the molecular pathogenesis of meningiomas (37), and Yang et al. identified *EPB41* as a hepatocellular carcinoma tumor suppressor that dysregulated in an allelic-specific fashion on the basis of functional-based assays *in vivo* and *in vitro* (38). Another factor interaction analysis for chromosome 8 and DNA methylation alterations highlighted that the *EPB41* family participates in innate immune response suppression and cytoskeletal changes in prostate cancer (39, 40). Together, this evidence supports the biological relevance of *EPB41* in tumor biology. Still, to date, the role of *EPB41* in OSA has not been reported.

To identify the two-gene signature, quantitative polymerase chain reaction was applied to test relative mRNA expression level of *PML* and *EPB41* in three human derived cell line, in which hFOB1.19 is the normal human osteoblast, while MG-63 and SAOS-2 are human osteosarcoma cell lines. *PML* and *EPB41* are likely to be down-regulated in aggressive tumors and linked with poor prognosis. Consistent results can be obtained on its expression in other tumor related researches mentioned above. Several limitations of the present study should be noted. The results suggest that the prognostic value of the *PML-EPB41* signature is independent of other clinical factors in OSA. However, the number of datasets needed to confirm this finding need to be expanded using GEO or Cancer Genome Atlas databases. Further integrated analysis may help to accurately predict the risk of OSA. If possible, the signature should be validated in collected clinical OSA biological samples. Secondly, no experimental data on the underlying mechanisms of *EPB41* in OSA have been obtained, and future additional well-designed experimental studies on *EPB41* will help define its functional role in OSA.

In summary, we established a 2-gene (*PML-EPB41*) signature that can be considered an innovative prognostic predictor for patients with OSA. This study provides new insights and novel molecular biomarkers for OSA prognosis, and the findings may help to discover novel therapeutic targets with clinical applications.

## Data Availability Statement

The data used for analysis during the current study are available from GEO public dataset and TARGET database.

## Author Contributions

SL, TS, and QF conceived and designed the study. SL and JL took a lead role in experiment and manuscript completion and generated the figures. SL collected the study data. SL and TS performed the genetic analyses. XY contributed his idea and supervised for improving research. All authors finalized the manuscript and approved the final manuscript.

### Conflict of Interest

The authors declare that the research was conducted in the absence of any commercial or financial relationships that could be construed as a potential conflict of interest.
