# Corrigendum: Identification of a Two-Gene (*PML-EPB41*) Signature With Independent Prognostic Value in Osteosarcoma

**DOI:** 10.3389/fonc.2021.671129

**Published:** 2021-04-14

**Authors:** Shengye Liu, Jiamei Liu, Xuechen Yu, Tao Shen, Qin Fu

**Affiliations:** ^1^Department of Spine and Joint Surgery, Shengjing Hospital of China Medical University, Shenyang, China; ^2^Department of Pathology, Shengjing Hospital of China Medical University, Shenyang, China; ^3^Hammer Health Sciences Center, Columbia University Medical Center, New York, NY, United States

**Keywords:** osteosarcoma, protein-protein interaction network, gene signature, prognostic prediction, survival analysis

In the original article, there was an error. **Dataset GSE39058 was miswritten as GSE39055, 42 samples was miswritten as 47**.

A correction has been made to ***RESULTS***, ***Identification of Seed Genes Based on the*** ***Coefficient of Variation in OSA***, ***1***:

In the present study, we established a 2-gene signature for the prognostic prediction of OSA **(Figure 1)**. First, we used the GSE39058 dataset, which included 47 samples, as a training set. In this dataset, each patient sample included detailed clinicopathologic information and survival status. The coefficient of variation (CV) of each probe was calculated for all samples, and the probes with a CV >20% were considered to have the largest degree of variation among all OSA samples and were selected as the seed probes. Then, 309 probes were obtained and mapped to 308 unique genes. Next, we completed an unsupervised clustering analysis of the 42 samples by using expression profiling of the 309 probes obtained in the previous step. As shown in **Figure 2A**, the OSA samples were divided into 2 groups, and there were significant differences in gene expression levels between the groups. Survival analysis was then used to compare the outcomes of the groups: no significant difference was observed between them (log-rank test *p* ≥ 0.05) **(Figure 2B)**.

The authors apologize for this error and state that this does not change the scientific conclusions of the article in any way. The original article has been updated.

